# Implementation status of maternal death surveillance and response system in Ethiopia: Evidence from a national-level system evaluation

**DOI:** 10.1371/journal.pone.0312958

**Published:** 2024-12-03

**Authors:** Neamin Tesfay, Alemu Zenebe, Zewdnesh Dejene, Henok Tadesse, Fitsum Woldeyohannes, Araya Gebreyesus, Amit Arora

**Affiliations:** 1 Ethiopian Public Health Institute, Centre of Public Health Emergency Management, Addis Ababa, Ethiopia; 2 Health Financing Program, Clinton Health Access Initiative, Addis Ababa, Ethiopia; 3 Department of Medical Microbiology and Immunology, College of Health Sciences, Mekelle University, Tigray, Ethiopia; 4 School of Health Sciences, Western Sydney University, Penrith, NSW, Australia; 5 Health Equity Laboratory, Campbelltown, NSW, Australia; 6 Translational Health Research Institute, Western Sydney University, Campbelltown, NSW, Australia; 7 Faculty of Medicine and Health, Discipline of Child and Adolescent Health, The Children’s Hospital at Westmead Clinical School, The University of Sydney, Westmead, NSW, Australia; 8 Oral Health Services, Sydney Local Health District and Sydney Dental Hospital, NSW Health, Surry Hills, NSW, Australia; The University of Hong Kong, CHINA

## Abstract

**Background:**

In Ethiopia, Maternal Death Surveillance and Response (MDSR) was integrated into the existing Integrated Disease Surveillance and Response (IDSR) system in 2014. Despite providing valuable evidence to inform policies and actions, system implementation has not been evaluated. Thus, a national-level evaluation was conducted to assess the level and status of system implementation.

**Methods:**

A national cross-sectional study was conducted using a multi-stage sampling approach in 2020. A total of 629 health facilities were included in the study. A modified tool, adapted from the World Health Organization (WHO) and the Centers for Disease Control and Prevention (CDC), was employed to assess each functional component of the system, encompassing structure, core, supportive, and system attributes. The score for each component was based on Ethiopian Public Health Institute’s mid-term evaluation metrics. To objectively evaluate the implementation status, a composite score of the Maternal Death Surveillance and Response Performance Index (MDSRPI) was calculated based on five performance indicators. Descriptive statistics, independent t-tests, and one-way analysis of variance (ANOVA) with Bonferroni correction were used to examine the variations in scores among the different characteristics.

**Results:**

Of the total sample size, 82.5% (519/629) of health facilities were assessed. Among the assessed health facilities, 77.0% (400/519) fulfilled the criteria for final analysis. Accordingly, the overall readiness score was 44.9% (95% CI: 43.9% to 45.9%), which is rated as less functional. The structures of the system were rated at 51.7% (95% CI: 49.9% to 53.4%), and the system attributes were rated at 69.6% (95% CI: 68.0% to 71.2%), which were considered fairly functional. In contrast, the core functions were rated at 20.0% (95% CI: 18.9% to 21.1%), and the supportive functions were rated at 38.4% (95% CI: 36.4% to 40.4%), which were categorized as not functioning and less functional, respectively. Regionally, Tigray’s overall readiness score (54.8%, 95% CI: 50.4–59.1%) was significantly higher than Oromia (41.6%, 95% CI: 40.2–43.0%, P = 0.0001), Amhara (47.7%, 95% CI: 43.9–45.9%, P = 0.05), and SNNPR (42.3%, 95% CI: 39.3–45.3, P = 0.0001). Additionally, Amhara’s score was significantly higher than Oromia and SNNPR. Secondary-level healthcare facilities (49.6%, 95% CI: 45.7–53.7, P = 0.029) had a significantly higher readiness score compared to primary health facilities (44.6%, 95% CI: 43.5–45.6). The overall score for the Maternal Death Surveillance and Response Performance Index (MDSPI) was 33.9%.

**Conclusion:**

Despite the noticeable regional variation, the overall system readiness and status to implement MDSR were suboptimal, characterized by low representativeness, completeness, and community engagement. Efforts should be directed toward improving community surveillance and enhancing all components of the system to address regional variations and improve overall performance through triangulation and integration with various data sources.

## Introduction

Public health surveillance is a tool used to estimate and monitor the health status and behavior of a population by implementing mechanisms for collecting, analysing, and interpreting data continuously, as well as disseminating information to those who need to take necessary actions [1–3]. Considering the paramount importance of surveillance in managing and containing large emerging and reemerging outbreaks, the WHO African Region designed and subsequently customized a strategy called Integrated Disease Surveillance and Response (IDSR) in 2000 [4]. IDSR is an all-rounded and evidence-based approach used to maximize national public health surveillance and response, commencing from the community to the national level [5].

In its inception, IDSR exclusively focused on infectious diseases, excluding other vital health events [6]. As the strategy evolved, significant experience was gained in implementing and effectively realizing it. Both essential human and financial resources have been dedicated to successfully integrating them into a country’s health system [7]. These tangible strides have created opportunities to consider other public health priorities, such as maternal death, to be included as mandatory notifiable events, which will be reported to subnational and national health authorities [8].

Considering the presence of an established surveillance platform and the limited availability of reliable mortality data sources (which are critical elements for the accurate estimation of mortality rates, including the maternal mortality rate [MMR]), such as vital event registration, which is capable of accurately quantifying and determining the causes of death, the importance of mortality surveillance has become increasingly prominent [9,10]. In addition, to accelerate the pace of reducing preventable maternal deaths globally, the United Nations Commission passed a resolution on the status of women in 2012 [11]. This resolution calls for the elimination of preventable maternal mortality through effective real-time measurements and tracking of death [12]. Thus, maternal death surveillance and response (MDSR) has become a model that works per ratified resolution [13].

MDSR is a continuous cycle of notification, review, and analysis of maternal deaths that could provide amicable solutions to prevent similar deaths in the future [14]. The system is expected to generate accurate and timely maternal mortality data (MMR tracking) and aims to identify major causes of maternal death, formulate appropriate interventions, and institute service delivery improvements. The response component is designed to act on quality of care improvement measures, community mobilization, and awareness [15–17].

At the national level, Ethiopia has accepted the initiative promoted by the WHO to bolster and redouble efforts to improve maternal health outcomes over the last three decades [18]. This is evidenced by a remarkable reduction in maternal mortality rates (MMR) from 871 per 100,000 live births (95% CI, 705–1039) in 2000 to 267 per 100,000 live births (95% CI, 189–427) in 2020, according to estimates made by the Ethiopian Demographic and Health Survey (EDHS), the World Health Organization (WHO), the United Nations Children’s Fund (UNICEF), the United Nations Population Fund (UNFPA), the World Bank Group, and the United Nations Department of Economic and Social Affairs (UNDESA) [19,20]. Accordingly, the system was piloted in 2013 in four larger agrarian regions and three city administrations, before being formally included in Ethiopia’s integrated disease surveillance structure, known as Public Health Emergency Management (PHEM), in 2014 [21,22]. In 2017, the perinatal death component was included, and the name of the system changed to Maternal and Perinatal Death Surveillance and Response (MPDSR) [23,24].

In Ethiopia, the system operates at two levels, a health facility, and a community, using the PHEM platform, which encompasses both indicator and event-based surveillance approaches [25]. The indicator-based approach involves the formal notification and reporting of maternal deaths that occur within the health tier system using a defined case definition through existing means of communication, extending from reporting health facilities to the national level. In contrast, event-based surveillance, typically seen as complementary to indicator-based surveillance, provides information on possible maternal deaths from various sources that often extend beyond the health system platform. Subsequently, the received information is scrutinized to determine whether it meets the criteria for reporting to the next level [26]. Notified deaths were investigated using verbal autopsy (VA) and Facility-Based Maternal Death Abstraction Format (FBMDAF) to facilitate the review. Subsequently, the investigated deaths were reviewed by the MPDSR committee, which is positioned at each reporting health facility, to determine the cause and contributing factor responsible for women’s deaths. Based on this review, an action plan will be developed to address the quality of care at health facilities, and the report will be sent to the national data hub using a structured format called the maternal death reporting format (MDRF) [27].

The Ethiopian government has disseminated the information generated from the system through policy briefs and the publication of annual performance reports, which are prepared with the involvement of the national technical working group for MPDSR [28]. In this regard, the system has made an indispensable contribution by providing ample evidence that plays a seminal role in promoting the use of non-pneumatic anti-shock garments (NASGs) for managing bleeding in women during referrals from health posts to higher facilities [29,30]. Furthermore, the evidence generated from the system has played a pivotal role in developing a national obstetric hemorrhage response plan, resulting in tangible progress such as the establishment of mini blood banks at Comprehensive Emergency Obstetric and Newborn Care (CEmONC) facilities [31,32]. Despite the system guiding evidence-based decisions, its implementation to the fullest extent was hampered by individual (lack of training, blame-shifting, and defensive attitudes), interpersonal (underreporting, lack of an effective feedback loop, and irregular supportive supervision), and organizational (limited engagement of the community, lack of reporting formats and guidelines, and inadequate available essential medical commodities) factors [33–39]. In addition to the aforementioned gaps in the system, there are limitations in working closely with Civil Registration and Vital Statistics systems (CRVS) in death identification and cause of death assignment, despite the CRVS system not being fully institutionalized [40–42]. Additionally, the slow pace of digitalization of the reporting mechanism and inadequate contextualization of the system in humanitarian settings were also identified as hindrances to advancing the system to the next level [43,44]. Thus, periodic evaluation is required to determine how well the system operates to meet its stated purposes and objectives. Accordingly, a national-level system evaluation was conducted to assess the level and extent of the MDSR system implementation.

## Methods and materials

### Study setting and period

Ethiopia is the second most populous country after Nigeria, with a population of 117 million in 2020 [45]. Furthermore, Ethiopia has nine regions and two city administrations, namely, Tigray, Afar, Amhara, Oromia, Somali, Benishangul-Gumuz, the Southern Nations Nationalities and Peoples Region (SNNPR), Gambella, Harari, the Addis Ababa city administration and the Dire Dawa city administration, before further balkanization of the SNNPR into four separate regions [46]. The country has 17187 health posts, 3724 health centres, 302 public hospitals, and 5401 private health facilities [47]. The evaluation was conducted at the national level from September 2019 to April 2020, after six years of system implementation.

### Study design, scope, and data collection tool

This nationwide cross-sectional study was conducted at selected health facilities, which included both hospitals and health centers. A modified version of the World Health Organization (WHO) and Centers for Disease Control and Prevention (CDC) evaluation tools was utilized [48,49]. The scope of the evaluation emphasized four components of surveillance systems: the structure of the system, core of function, supportive function, and system attributes. The structure of the system component primarily aimed to assess the presence of mandatory notifications, coordinating bodies, strengthening strategies, and networks and partnerships. On the other hand, the component included under the core function of the system examines the process and output of the system, which extends from case detection to feedback. Furthermore, the supportive function component comprehensively overlooks the availability of a conducive environment for the smooth implementation of the system. This includes focusing on the availability of resources, means of communication, training, supportive supervision, standards, and guidelines. Finally, the quality of surveillance was evaluated using both qualitative and quantitative indicators of system attributes. Simplicity, flexibility, acceptability, usefulness, and stability were qualitative indicators of system quality, while review rate and community engagement were quantity indicators used to objectively assess the quality of the surveillance. A diagrammatic representation of each component and their definitions are provided in the **S1 and S2 Appendices in S1 File**. Furthermore, the modified data collection tool and its respective scores are provided in **S3 Appendix in S1 File.**

### Sample size determination, sampling strategy, and sample allocation

Multistage sampling techniques were used to select the health facilities for the study. The study population (health centers and hospitals) was nested within districts, with districts further nested within zones (provinces) and provinces nested into regions. The design effect was included in the sample size estimation to accommodate for the clustering effect and gain power. Accordingly, the sample size was determined first, and all regions were selected as primary sampling units because of their small size. Second, a random sample of health facilities and hospitals was selected using stratified random sampling techniques, based on the type of facilities as stratifying variables. To determine the sample size *N* = *N*_1_+*N*_2_, where N_1_ is the total number of health centres and N_2_ is the total number of hospitals under investigation in Ethiopia. The critical value of the standard normal distribution (Z_α/2_), the pooled variance of system performance based on the indicators to measure the system (*σ*^2^), must be less than a given accuracy or marginal error (ϵ), i.e., $\frac{Z_{\alpha /2}*\sigma }{\sqrt{n}}<\epsilon$. Furthermore, the design effect (deff) was computed using this formula *Deff* = 1+(*m*−1)*ρ [50], where ρ is intercorrelated with the cluster due to the similarity of observation within the region rather than between regions—a value typically between 0 and 1 (common conservative choice is 0.33), with m being the average number of observations within clusters [regions] consisting of health centres or hospitals. The computed deff value of 1.2 aligns with the number of health studies [51,52]. The required sample size, N = N_1_ [sample of health center] + N_2_ [sample of hospitals], was computed based on the relevant parameters.

Thus, the final sample size for the study based on the parameters was computed as:

$$n_{i}>\left[\frac{Z_{\frac{\alpha }{2}}^{2}*\sigma^{2}+\epsilon^{2}}{\epsilon^{2}+\frac{Z_{\frac{\alpha }{2}}^{2}*\sigma^{2}}{N_{i}}}\right]*Deff\;i=1,2\;for\;hospitals\;and\;health\;facilities$$

Or it can be simplified as

$n_{0i}>\left[\frac{Z_{\frac{\alpha }{2}}^{2}*\sigma^{2}}{\epsilon^{2}}\right]$ is the initial population size, but the study population size is known; then, the required sample size for *i*^*th*^ strata was computed using *n*_0*i*_ and *N*_*i*_ as follows:

$n_{i}=\frac{n_{0i}*N_{i}}{n_{oi}+N_{i}-1},i=1,2$ denoting the sample of health center size or hospital size determined. Thus, the sample size is computed based on the available parameters using the above formula:

$$n_{health\;centers}=552\;n_{hospitals}=77$$

The total sample size was computed as $n=n_{health\;centers}+n_{hospitals}=629$, and other detailed explanations of the sample size determination are provided in the **S4 Appendix in S1 File.** Moreover, sample allocation was based on the probability proportional to their size [PPS] for each region, specifically using $\frac{n}{N}=\frac{n_{r}}{N_{r}}$. Then, the sample allocated for each region was computed with $n_{r}=\frac{n}{N}*N_{r}$, where *n*_*r*_ is the sample size allocated for each region, *N*_*r*_ is the total number of health centers or hospitals for *r*^*th*^ *r* = 1,2,…,11, and *N* is the total number of facility types under investigation.

### Data sources and survey participants

Facility-level information was obtained from MDSR focal points and medical directors of the health facilities. The evaluation was conducted in public health facilities, which account for 13% of the total public facilities (i.e., only including health centers and hospitals), by excluding private, non-governmental organizations (NGOs), and special clinics.

### Data collection tools and quality management

The data collection tool was tested with 19 selected health facilities (seven hospitals and 12 health centers), and necessary corrections were made to the tool before it was used for full-scale data collection. Subsequently, the data collectors underwent a three-day training session and were provided with a field guide and a nearby field supervisor. The entire data collection process was conducted using an open data kit (ODK), and the quality of the data was checked and corrected with the help of the central teams.

### Data management and analysis

The readiness of the facilities to fully implement the system was measured using four dimensions (structure, core, support, and attributes) of the surveillance component. Before calculating the single-weight average readiness score, the scores for each dimension of the system were independently computed. Subsequently, by equally weighting the dimensions, a single weighted measure of health facility readiness was computed, as shown in **S5 Appendix in S1 File**.

### Dimension score measurement

1. System structure: This component was measured using seven variables with yes/no responses. The overall score for the structural system (S_T_) was computed and weighted accordingly to produce the final weighted score (S_W_)_._
2. Core function: This component was assessed using 11 variables with yes or no responses. The overall score for core function (C_T_) was calculated, and the weight score (C_W)_ was computed.
3. Supportive function: This dimension was gauged using ten variables with a yes/no response. The total supportive function (SU_T_) score was calculated based on the total score and the weighted score (SU_W_) was calculated.
4. System attributes: Five subcategories with 26 items (simplicity (7), flexibility (2), acceptability (5), usefulness (8), and stability (4)) with yes/no responses were used to compute the overall score (A_T_). The weight score (A_W_) was then calculated.
5. Overall score: To compute the overall score (OT), the weighted score of each dimension was summed to produce the final score. Subsequently, all scores in each dimension, including the overall score, are presented using the mean ($\tilde{x}$) and standard deviation (SD) after confirming the skewness levels of the observations. Furthermore, after assessing normality using the Shapiro-Wilk test, independent sample t-tests (for two-group variables) and one-way analysis of variance (ANOVA) were employed to examine the variation in the scores across various features. In cases where ANOVAs were significant, Bonferroni post-hoc analyses were used to identify further mean differences. The level of statistical significance was set at a two-sided p-value of < 0.05. Additionally, the overall score, including the score for each component, was rated according to the guidelines of the midterm evaluation of the Ethiopian Public Health Institutes (EPHI). The ratings are as follows: ’91 to 100% = Very effectively functioning = fully achieved, very few or no shortcomings’, ’76 to 90% = effectively functioning = largely achieved, despite a few shortcomings’, ’51 to 75% = Fairly functioning = only partially achieved’, ’21 to 50% = Less functioning = very limited achievement, extensive shortcomings, and ’20% or less = Not functioning = not achieved’ [53].

#### Measurement of maternal death surveillance and response performance index

To objectively assess the status of system implementation, five parameters were used to provide a comprehensive picture and gauge the system over the years of implementation [28]. Accordingly, notification rate (R_n_), notification coverage rate (CR_n_), review rate (R_r_), review coverage rate (CR_r_), and proportion of reviewed community deaths (CRD_p_) were computed to derive the Maternal Death Surveillance and Response Performance Index (MDSRPI). All these parameters were calculated by summing each numerator and denominator over a seven-year timeframe from 2014 to 2020.

A. Maternal death notification rate

The notification rate (R_n_) was computed to assess how well the system is capturing and notifying deaths using potential data sources at both the health facility and community levels. In Ethiopia, every death should be reported to the next level on a weekly basis through the Public Health Emergency Management (PHEM) system. A score below 80% indicates a gap in the effective notification of deaths. To calculate the R_n_ score, the number of weekly notified deaths (d_n_) from 2014 to 2020 was taken as the numerator, and the identified deaths (d_i_) from potential data sources, such as the District Health Information System (DHIS_2) and performance reports, from 2014 to 2020 were considered as the denominator. The details are depicted in Table 1.

B. Maternal death notification coverage rate

The maternal death notification coverage rate (CR_n_) was calculated to assess how well the system captures estimated(excepted) deaths, which is foundational for measuring the representativeness of the system. Accordingly, the number of notified deaths (d_n_) reported from 2014 to 2020 was taken as the numerator, and the estimated deaths (E(d)) were taken as the denominator. The E(d) was measured using the UN Maternal Mortality Estimation Inter-Agency Group (UN MMEIG) estimation and the Ethiopian population projections from 2014 to 2020 based on the 2007 census [54,55]. The details are depicted in Table 1.

C. Maternal death review coverage rate

The maternal death review coverage rate (CR_r_) is a parameter used to assess the status of the Maternal Death Surveillance and Response (MDSR) system. It reflects the capacity to review deaths that occurred at both community and facility levels, in comparison to the expected deaths (E(d)). CR_r_ is computed using the number of deaths reviewed (d_r_) that are reported to the central data hub using the Maternal Death Reporting Format (MDRF) during the years 2014 to 2020. These deaths are investigated and extracted using Verbal Autopsy (VA) for community-level deaths and the Facility-Based Maternal Death Abstraction Format (FBMDAF) for facility deaths before being reported using MDRF. The denominator is the expected deaths (E(d)), and the details are depicted in Table 1.

D. Maternal death review rate

The maternal death review rate (R_r_) is an indicator used to provide insight into how capable the system is at reviewing deaths after notification using the PHEM weekly reporting system. A score above 100% indicates that deaths are not being notified in consideration of potential data sources (i.e., various points of care in health facilities). To compute the R_r_ score, d_n_ was taken as the denominator for deaths notified from 2014 to 2020, and d_r_ as the numerator for deaths reviewed within the same period.

E. Proportion of reviewed community deaths

The proportion of reviewed community deaths (CRD_P_) is used as a resolution to effectively monitor the community loop of the surveillance. To calculate CRD_P_, deaths reported through MDRF, investigated, and extracted using VA were declared as community deaths (d_rc_) and served as the numerator, while d_r_ was used as the denominator to compute the proportion. The details are depicted in Table 1. In addition, the Mann–Kendall trend test and Sen’s slope were used to assess the trend in the utilization of various data sources for maternal death reviews over the year [56].

**Table 1:** Key performance indicators to measure the status of maternal death surveillance in Ethiopia from 2014 to 2020.

		Notification		Review	
	Notification rate	Notification coverage rate	Review rate	Review coverage rate	Proportion reviewed community deaths
**Definition**	$\sum_{i=2014}^{2020}R_{n}=\frac{d_{n}}{d_{i}}$	$\sum_{i=2014}^{2020}{CR}_{n}=\frac{d_{n}}{E(d)}$	$\sum_{i=2014}^{2020}R_{r}=\frac{d_{r}}{d_{n}}$	$\sum_{i=2014}^{2020}{CR}_{r}=\frac{d_{r}}{E(d)}$	$\sum_{i=2014}^{2020}{CRD}_{P}=\frac{d_{rc}}{d_{r}}$
**Data source for numerator**	Deaths notified to central level ^1^	Deaths notified to central level ^1^	Deaths with reviews reported to central level ^3^	Deaths with reviews reported to central level ^3^	Deaths with reviews reported from the community to the central level ^4^
**Data source for denominator**	Deaths identified from other sources ^2^	Death estimated ^5^	Deaths notified to central level ^1^	Deaths estimated ^5^	Deaths with reviews reported to central level ^3^

R_n_ = notification rate.

R_r_ = review rate.

CR_n_ = notification coverage rate.

CR_r_ = review coverage rate.

d_i_ = deaths identified.

d_n_ = deaths notified.

d_r_ = deaths reviewed.

d_rc_ = reviewed community deaths.

CRD_P_ = Proportion of reviewed community deaths.

E(d) = estimated deaths.

^1^: Deaths notified through the PHEM weekly reporting system from 2014 to 2020.

^2^: Deaths identified from potential other sources (DHIS 2 reports and facility-level registries) from 2014 to 2020.

^3^: Deaths reviewed using both data sources (Verbal Autopsy (VA) and the Facility-Based Maternal Death Abstraction Format (FBMDAF) from 2014 to 2020 were sent to the national data hub using the Maternal Death Report Format (MDRF).

^4:^ Deaths are reviewed at the community level and sent to the national data hub using the Maternal Death Report Format (MDRF) after extracting information through Verbal Autopsy (VA) from 2014 to 2020.

^5:^ Deaths estimated based on population projection from 2007 and considering the UN MMEIG 2017 report.

After computing each of the five parameters, the composite score of the MDSPI was calculated to provide a judgment on the overall status of the system. The weighting of the aforementioned parameters was conducted in consideration of their role in defining the system, in consultation with the national technical working group for MPDSR. CR_n_ and CR_r_ were weighted 0.3 each out of 1 due to their role in defining the representativeness of the system. Similarly, R_r_ was weighted 0.2 due to its indication of the capacity of the review committee to review and set actions to mitigate similar future deaths. R_n_ and CRD_P_ were weighted 0.1 each because they show the level of completeness and community loop of the surveillance. After computing each score per the defined weighting criteria, the MDSRPI was produced by summing each weighted indicator. Subsequently, the MDSRPI was further classified into three groups: a score below 0.4 was considered “low performing,” a score between 0.4 and 0.6 was declared “moderate performing,” and a score above 0.6 was declared “good performing” [57].

Furthermore, health facilities were classified into three categories, primary, secondary, and tertiary, based on their human resources and the types of services they provide. Additionally, the health facilities were classified into five altitude groups: desert areas (below 500 meters), lowlands (500–1500 meters), midlands (1500–2300 meters), highlands (2300–3200 meters), and upper highlands (3200 meters and above) [58]. Moreover, the regions were classified into three contextual categories—pastoralist, agrarian, and city administration–based on the cultural and socioeconomic backgrounds of their populations [59].

Overall, the data were exported from ODK to STATA version 17 for recoding, cleaning, and statistical analysis. According to the preliminary analysis, data were collected from 519 health facilities (445 health centers and 74 hospitals). Among them, 400 health facilities were categorized as MDSR-implementing facilities because they had notified and reviewed at least one maternal death a year before the survey [60]. This study was exclusively focused on these facilities.

### Ethical statement

The assessment protocol received ethical clearance from the Ethiopian Public Health Institute Institutional Review Board (EPHI-IRB) with reference no. EPHI 5.3_1/36. A support letter from the EPHI was also written to all selected woredas, regional health bureaus, and selected health facilities, indicating the objective and final aim of the evaluation. Informed verbal consent was also obtained from the study participants by informing them about the evaluation and purpose of the study. Each respondent was allowed to provide consent or refuse to participate after receiving complete information regarding the study. The data collection period spanned from 20/02/2020 to 20/06/2020, and all records, including medical records, were fully anonymized before being accessed for this study. This process was aligned with IRB requirements, which mandate full anonymity.

## Results

### Characteristics of the MDSR implementing facilities

Of the 400 MDSR implementing facilities, primary healthcare facilities accounted for 89.3%, while secondary health facilities contributed 7.3% to the total. Furthermore, 78.5% of the facilities had implemented the system for more than two years, and 59.5% of them were in rural areas. Regionally, 70.5% of the health facilities were located in the Oromia and Amhara regions. According to the agroecological zone classification, more than half (51.8%) were located in midland areas (Table 2).

**Table 2:** Characteristics of MDSR implementing health facilities in Ethiopia, 2020 (N = 400).

Characteristic	Category	Frequency	Percentage (%)
**Type of health facility**			
	Primary healthcare level	357	89.3
	Secondary healthcare level	29	7.3
	Tertiary healthcare level	14	3.5
**Year of implementation**			
	Less than <2	86	21.5
	More than≥2	314	78.5
**Region**			
	Addis Ababa	17	4.3
	Amhara	112	28.0
	Afar	2	0.5
	Benishangul Gumuz	10	2.5
	Dire Dawa	4	1.0
	Gambella	4	1.0
	Harari	2	0.5
	Oromia	170	42.5
	SNNPR	52	13.0
	Somali	2	0.5
	Tigray	25	6.3
**Location of health facility**			
	Rural	238	59.5
	Urban	162	40.5
**Type of region**			
	Agrarian	359	89.8
	City administration	23	5.8
	Pastoralist	18	4.4
**Agroecological zone**			
	Dessert	6	1.5
	Lowland	57	14.3
	Midland	207	51.8
	Highland	127	31.8
	Upper highland	3	0.8

### Health facility readiness for the implementation of maternal death surveillance and response

The national mean score for the system structure was 51.7% (95% CI: 49.9% to 53.4%). The mean score for secondary health facilities was 63.1% (95% CI: 57.1% to 69.1%), which was significantly higher than the mean score of primary health facilities at 50.4% (95% CI: 48.6% to 52.2%, P ≤ 0.0001). Regionally, the mean score for the Tigray region was 66.3% (95% CI: 58.7% to 73.9%), which is noticeably higher than the mean scores for Oromia at 46.7% (95% CI: 44.2% to 49.9%, P ≤ 0.001), SNNPR at 51.6% (95% CI: 46.5% to 56.8%, P = 0.02), and Amhara at 52.2% (95% CI: 49.2% to 55.1%, P = 0.007). Urban health facilities scored higher at 55.3% (95% CI: 52.8%–57.8%) than rural facilities at 49.3% (95% CI: 47.0%–51.6%, P = 0.0007) (Table 3).

**Table 3:** Mean ($\tilde{x}$) and standard error (SE) scores for the structure of the system among MDSR implementing health facilities in Ethiopia, 2020 (n = 400).

Structure of the system							
Characteristic	Category	Over all	$\tilde{x}$ (SE) (%)	95%CI	Comparison	Absolute Difference (%)	P Value
**Type of health facility**	** **	** **					
	Primary healthcare level	357	50.4 [0.91]	[48.6 to 52.2]	_	_	_
	Secondary healthcare level	29	63.1[2.97]	[57.1 to 69.1]	Secondary vs Primary	12.6	0.0001
	Tertiary healthcare level	14	61.2[4.35]	[51.8 to 70.6]	_	_	_
**Year of implementation**							
	Less than<2	86	51.9[1.72]	[48.6 to 55.4]	_	_	_
	More than ≥2	314	51.6[1.01]	[49.6 to 53.6]	_	_	_
**Region**							
	Addis Ababa	17	66.4[2.72]	[60.6 to 72.2]	Addis Ababa Vs Oromia	19.7	0.0001
	Amhara	112	52.2[1.48]	[49.2 to 55.1]	Amhara Vs Tigray	14.1	0.007
	Afar	2	42.9[0.0]	NA	_	_	_
	Benishangul Gumuz	10	58.6[4.49]	[48.4 to 68.7]	_	_	_
	Dire Dawa	4	57.1[5.83]	[38.6 to 75.7]	_	_	_
	Gambella	4	71.4 [5.83]	[52.9 to 90.0]	_	_	_
	Harari	2	42.9 [14.29]	NA	_	_	_
	Oromia	170	46.7 [1.29]	[44.2 to 49.3]	Oromia Vs Tigray	19.5	0.0001
	SNNPR	52	51.6[2.55]	[46.5 to 56.8]	_	_	_
	Somali	2	78.6[7.14]		_	_	_
	Tigray	25	66.3[4.68]	[58.7 to 73.9]	Tigray Vs SNNPR	14.6	0.016
**Location of health facility**					_	_	_
	Rural	238	49.3[1.17]	[47.0 to 51.6]	_	_	_
	Urban	162	55.3 [1.26]	[52.8 to 57.8]	Urban Vs Rural	6.0	0.0007
**Agroecological zone**							
	Dessert	6	66.7[6.02]	[51.2 to 82.2]	_	_	_
	Lowland	57	53.4[2.10]	[49.2 to 57.6]	_	_	_
	Midland	207	51.3[1.31]	[48.7 to 53.9]	_	_	_
	Highland	127	51.1[1.41]	[48.3 to 53.9]	_	_	_
	Upper highland	3	47.6[9.52]	[6.6 to 88.6]	_	_	_
**National level score**		400	51.7[0.88]^c^	[49.9 to 53.4]	_	_	_

• ^c^: fairly functioning.

• Variables with significant variation based on the respective characteristic (based on an independent t-test or one-way ANOVA with subsequent Bonferroni post-analyses) are displayed.

• ‘-‘Having insignificant variation.

The national mean score for the core function was 20.0% (95% CI: 18.9% to 22.1%). The Tigray region scored significantly better at 29.8% (95% CI: 25.0% to 34.6%) compared to Oromia at 16.7% (95% CI: 15.4% to 18.1%, P ≤ 0.0001), SNNPR at 14.5% (95% CI: 12.8% to 16.2%, P ≤ 0.0001), and Addis Ababa at 17.1% (95% CI: 11.8% to 22.4%, P = 0.004). Furthermore, the score for the core function in the Amhara region was 25.2% (95% CI: 23.1% to 27.3%), which was significantly higher than that of Oromia and SNNPR. Similarly, the Benishangul Gumuz region had a significantly greater score of 26.4% (95% CI: 18.6% to 34.1%) compared to the SNNPR region (P = 0.04) (Table 4).

**Table 4:** Mean ($\tilde{x}$) and standard error (SE)scores core function of the system among MDSR implementing health facilities in Ethiopia, 2020 (n = 400).

The core function of the system							
Characteristic	Category	Over all	$\tilde{x}$ (SE) (%)	95%CI	Comparison	Absolute Difference (%)	P_ Value
Type of health facility		** **					
	Primary healthcare level	357	20.5[0.59]	[19.3 to 21.6]	_	_	_
	Secondary healthcare level	29	18.2[2.12]	[14.0 to 22.3]	_	_	_
	Tertiary healthcare level	14	14.3[2.79]	[8.4 to 20.1]	_	_	_
Year of implementation							
	Less than<2	86	18.5[1.08]	[16.4 to 20.6]	_	_	_
	More than ≥2	314	20.5[0.65]	[19.3 to 21.8]	_	_	_
Region							
	Addis Ababa	17	17.1[2.69]	[11.8 to 22.4]	Addis Ababa Vs Tigray	12.7	0.004
	Amhara	112	25.2[1.06]	[23.1 to 27.3]	Amhara Vs Oromia	8.4	0.0001
	Afar	2	18.2[9.09]	[0.3 to 36.1]			
	Benishangul Gumuz	10	26.4[3.94]	[18.6 to 34.1]	Benishangul Gumuz Vs SNNPR	11.9	0.041
	Dire Dawa	4	31.8[2.62]	[26.7 to 37.0]	_	_	_
	Gambella	4	18.2[6.43]	[5.5 to 30.8]	_	_	_
	Harari	2	27.3[0.0]	NA			
	Oromia	170	16.7[0.68]	[15.4 to 18.1]	Oromia Vs Tigray	13.1	0.0001
	SNNPR	52	14.5[1.35]	[11.9 to 17.2]	SNNPR vs Amhara	10.7	0.0001
	Somali	2	13.6[4.55]	[4.7 to 22.6]			
	Tigray	25	29.8[2.43]	[25.0 to 34.6]	Tigray Vs SNNPR	15.3	0.0001
Location of health facility							
	Rural	238	20.9[0.70]	[19.6 to 22.3]	_	_	_
	Urban	162	18.9[0.92]	[17.1 to 20.7]	_	_	_
Agroecological zone							
	Dessert	6	18.2[4.69]	[9.0 to 27.4]	_	_	_
	Lowland	57	21.7[1.47]	[18.8 to 24.6]	_	_	_
	Midland	207	19.6[0.76]	[18.1 to 21.3]	_	_	_
	Highland	127	20.0[1.03]	[18.0 to 22.1]	_	_	_
	Upper highland	3	27.3[5.25]	[17.0 to 37.6]	_	_	_
National level		400	20.0[0.56] ^a^	[18.9 to 21.1]	_	_	_

• ^a^: not functioning.

• Variable with significant variation based on the respective characteristic (based on an independent t-test or one-way ANOVA with subsequent Bonferroni post-analyses) are displayed.

• ‘-‘Having insignificant.

On the other hand, the national mean score for supportive functions was 38.4% (95% CI: 36.4% to 40.4%). The mean score for secondary health facilities was 50.2% (95% CI: 41.0% to 59.4%), which was significantly greater than that for primary health facilities at 37.3% (95% CI: 35.2% to 39.3%, P = 0.003). Regionally, the mean score for the Tigray region was 54.2% (95% CI: 45.6% to 62.8%), which was significantly greater than that for Oromia at 35.7% (95% CI: 33.0% to 38.5%, P = 0.001) and SNNPR at 31.3% (95% CI: 24.9% to 37.7%, P ≤ 0.0001) (Table 5).

**Table 5:** Mean ($\tilde{x}$) and standard error (SE) supportive function of the system among MDSR implementing health facilities in Ethiopia, 2020 (n = 400).

Supportive function of the system							
Characteristic	Category	Overall	$\tilde{x}$ (SE) (%)	95%CI	Comparison	Absolute Difference (%)	P value
Type of health facility							
	Primary healthcare level	357	37.3[0.10]	[35.2 to 39.3]	_	_	_
	Secondary healthcare level	29	50.2[4.7]	[41.0 to 59.4]	Secondary Vs Primary	12.9	0.003
	Tertiary healthcare level	14	44.2[5.3]	[33.7 to 54.6]	_	_	_
Year of implementation							
	Less than<2	86	37.1[2.26]	[32.7 to 41.5]	_	_	_
	More than ≥2	314	38.8[1.14]	[36.6 to 41.0]	_	_	_
Region							
	Addis Ababa	17	36.9[3.44]	[30.1 to 43.7]	_	_	_
	Amhara	112	39.9[0.02]	[36.3 to 43.4]	_	_	_
	Afar	2	59.1[13.6]	[32.3 to 85.9]	_	_	_
	Benishangul Gumuz	10	50.0[6.8]	[36.6 to 63.4]	_	_	_
	Dire Dawa	4	63.6[3.71]	[45.3 to 70.9]	_	_	_
	Gambella	4	43.2[15.9]	[11.9 to 74.5]	_	_	_
	Harari	2	50.0[22.7]	[5.3 to 94.7]	_	_	_
	Oromia	170	35.7[1.40]	[33.0 to 38.5]	_	_	_
	SNNPR	52	31.3[3.26]	[24.9 to 37.7]	SNNPR Vs Tigray	22.9	0.00001
	Somali	2	40.9[4.55]	[32.0 to 49.8]	_	_	_
	Tigray	25	54.2[4.37]	[45.6 to 62.8]	Tigray Vs Oromia	18.5	0.001
Location of health facility					_	_	_
	Rural	238	37.4[1.27]	[34.9 to 39.8]	_	_	_
	Urban	162	40.0[1.67]	[36.7 to 43.3]	_	_	_
Agroecological zone							
	Dessert	6	42.4[13.00]	[16.9 to 68.0]	_	_	_
	Lowland	57	40.5[2.93]	[34.7 to 46.3]	_	_	_
	Midland	207	38.6[1.40]	[35.9 to 41.4]	_	_	_
	Highland	127	36.9[1.72]	[33.5 to 40.2]	_	_	_
	Upper highland	3	45.5[5.25]	[35.1 to 55.8]	_	_	_
National level		400	38.4[1.01] ^b^	[36.4 to 40.4]	_	_	_

• ^b:^ less functioning.

• Variable with significant variation based on the respective characteristic (based on an independent t-test or one-way ANOVA with subsequent Bonferroni post-analyses) are displayed.

• ‘-‘Having insignificant variation.

Furthermore, the national mean score for system attributes was 69.6% (95% CI: 68.0% to 71.2%). The mean score for the Amhara region was 73.7% (95% CI: 70.8% to 76.5%), which was significantly greater than that for the Oromia region at 67.1% (95% CI: 64.6% to 69.6%, P = 0.04) (Table 6). Overall, the national readiness score for MDSR implementation was 44.9% (95% CI: 43.9% to 45.9%). The mean score for secondary health facilities was 49.7% (95% CI: 45.7% to 53.7%), which was significantly greater than that for primary health facilities at 44.6% (95% CI: 43.5% to 45.6%, P = 0.03). Regionally, the mean score for the Tigray region was 54.8% (95% CI: 50.4% to 59.1%), which was significantly greater than that for Oromia at 41.6% (95% CI: 40.2% to 43.0%, P ≤ 0.0001), Amhara at 47.7% (95% CI: 46.0% to 49.4%, P = 0.05), and SNNPR at 42.3% (95% CI: 39.3% to 45.0%, P ≤ 0.0001). Similarly, the mean score for the Amhara region was significantly higher than that for the Oromia region (P ≤ 0.0001) and SNNPR (P = 0.04) (Table 7).

**Table 6:** Mean ($\tilde{x}$) and standard error (SE) score for the attribute of the system among MDSR implementing health facilities in Ethiopia, 2020 (n = 400).

System attribute							
Characteristic	Category	Overall	$\tilde{x}$ (SE)(%)	95%CI	Comparison	Absolute Difference (%)	P_ Value
Type of health facility							
	Primary healthcare level	357	70.1[0.85]	[68.4 to 71.8]	_	_	_
	Secondary healthcare level	29	67.4[3.20]	[61.1 to 73.7]	_	_	_
	Tertiary healthcare level	14	63.3[4.38]	[54.7 to 71.9]	_	_	_
Year of implementation							
	Less than<2	86	71.0[1.69]	[67.7 to 74.3]	_	_	_
	More than ≥2	314	69.3[0.93]	[67.5 to 71.1]	_	_	_
Region							
	Addis Ababa	17	62.3[3.31]	[55.8 to 68.8]	_	_	_
	Amhara	112	73.7 [0.01]	[70.8 to 76.5]	_	_	_
	Afar	2	45.2[19.3]	[7.3 to 83.2]	_	_	_
	Benishangul Gumuz	10	72.4[4.95]	[62.6 to 82.1]	_	_	_
	Dire Dawa	4	71.5[9.00]	[53.8 to 89.2]	_	_	_
	Gambella	4	84.0[5.26]	[73.7 to 94.3]	_	_	_
	Harari	2	57.1[5.83]	[45.7 to 68.6]	_	_	_
	Oromia	170	67.1[1.26]	[64.7 to 69.6]	Oromia Vs Amhara	6.5	0.045
	SNNPR	52	71.8[2.39]	[67.1 to 76.5]	_	_	_
	Somali	2	67.9[3.10]	[61.8 to 73.9]	_	_	_
	Tigray	25	68.8[2.79]	[63.3 to 74.3]	_	_	_
Location of health facility							
	Rural	238	70.0[1.06]	[67.9 to 72.1]	_	_	_
	Urban	162	69.2[1.26]	[66.7 to 71.6]	_	_	_
Agroecological zone							
	Dessert	6	77.3[5.37]	[66.8 to 87.9]	_	_	_
	Lowland	57	69.7[2.21]	[65.4 to 74.1]	_	_	_
	Midland	207	68.9[1.16]	[66.6 to 71.2]	_	_	_
	Highland	127	70.2[1.38]	[67.5 to 72.9]	_	_	_
	Upper highland	3	80.4[6.56]	[67.5 to 93.3]	_	_	_
National level		400	69.6[0.81]^c^	[68.0 to 71.2]	_	_	_

• ^c:^ fairly functioning.

• Variable with significant variation based on the respective characteristic (based on an independent t-test or one-way ANOVA with subsequent Bonferroni post-analyses) are displayed.

• ‘- ‘Having insignificant variation.

**Table 7:** Mean ($\tilde{x}$) and standard error (SE) scores for overall health facility readiness among MDSR implementing health facilities in Ethiopia, 2020 (n = 400).

Overall readiness attribute							
Characteristic	Category	Overall	$\tilde{x}$ (SE)(%)	95%CI	Comparison	Absolute Difference (%)	P Value
Type of health facility							
	Primary healthcare level	357	44.6[0.54]	[43. 5 to 45.6]	_	_	_
	Secondary healthcare level	29	49.7[2.05]	[45.7 to 53.7]	Secondary Vs Primary	5.1	0.029
	Tertiary healthcare level	14	45.7[2.55]	[40.7 to 50.8]	_	_	_
Year of implementation							
	Less than<2	86	44.7[1.07]	[42.5 to 46.8]	_	_	_
	More than ≥2	314	45.1[0.59]	[43.9 to 46.2]	_	_	_
Region							
	Addis Ababa	17	45.7[1.99]	[41.8 to 49.6]	_	_	_
	Amhara	112	47.7[0.01]	[46.0 to 49.4]	Amhara Vs Tigray	7.1	0.05
	Afar	2	41.3[10.50]	[20.7 to 62.0]	_	_	_
	Benishangul Gumuz	10	51.8[2.55]	[46.8 to 56.8]	_	_	_
	Dire Dawa	4	56.0[3.02]	[50.1 to 62.0]	_	_	_
	Gambella	4	54.2[4.51]	[45.3 to 63.1]	_	_	_
	Harari	2	44.3[7.79]	[29.0 to 59.6]	_	_	_
	Oromia	170	41.6[0.72]	[40.2 to 43.0]	Oromia vs Amhara	6.1	0.0001
	SNNPR	52	42.3[1.52]	[39.3 to 45.3]	SNNPR vs Amhara	5.4	0.04
	Somali	2	50.2[2.56]	[45.2 to 55.3]	_	_	_
	Tigray	25	54.8[2.21]	[50.4 to 59.1]	[Tigray vs Oromia]^1^ [Tigray vs SNNPR]^2^	[13.2]^1^ [12.5]^2^	0.0001
Location of health facility							
	Rural	238	44.4[0.67]	[43.1 to 45.7]	_	_	_
	Urban	162	45.8[0.81]	[44.2 to 47.4]	_	_	_
Agroecological zone							
	Dessert	6	51.1[3.86]	[43.6 to 58.7]	_	_	_
	Lowland	57	46.3[1.27]	[43.8 to 48.8]	_	_	_
	Midland	207	44.6[0.76]	[43.1 to 46.1]	_	_	_
	Highland	127	44.5[0.86]	[42.9 to 46.2]	_	_	_
	Upper highland	3	50.2[3.96]	[42.4 to 58.0]	_	_	_
National level		400	44.9[0.52] ^b^	[43.9 to 45.9]	_	_	_

• ^b^: less functioning: Variable with significant variation based on the respective characteristic (based on an independent t-test or one-way ANOVA with subsequent Bonferroni post analyses) are displayed: ‘- ‘Having insignificant variation.

• ^1^ [Tigray vs Oromia] with a difference of 13.2%, P value = 0.0001: ^2^[Tigray vs SNNP] with a difference of 12.5%, P value = 0.0001.

### Maternal death surveillance and response system performance indictors

The notification rate was 76.3%, with a minimum of 44.3% from the Tigray region and a maximum of 85.3% from the Amhara region. Since the score was below 80%, it indicates a gap in completeness and the need for exhaustively searching for potential data sources before notifying the next level. Furthermore, both the coverage rate for notification and review were below 10%, clearly demonstrating that the system has issues depicting the real picture and is not in a position to estimate the magnitude, which is one of its objectives. However, Harir and Dire Dawa have better notification and review coverage rates due to their proximity to the Oromia and Somalia regions and their tertiary facilities serving as catchment areas. This results in a case overload, potentially inflating performance metrics. However, the proportion of reviewed community deaths, typically tracked and reviewed for their permanent residents, is below 20%, indicating that most reviewed deaths are from nearby regions (Table 8).

**Table 8:** Summary of key maternal death surveillance and response performance indicators in Ethiopia from 2014 to 2020.

Region	Expected death	# of identified deaths (all sources)	# of Notified death	Reviewed deaths	# of Reviewed community death	Reviewed Facility death	Notification rate (%)	Notification coverage rate (%)	Review coverage rate (%)	Review rate (%)	Proportion of reviewed community deaths (%)	MDSRPI score (%)
Addis Ababa	2268	414	340	274	49	225	82.1	15.0	12.1	80.6	17.9	34.2
Afar	1273	188	87	78	41	37	46.3	6.8	6.1	89.7	52.6	31.7
Amhara	10552	1707	1456	1250	836	414	85.3	13.8	11.8	85.9	66.9	40.1
Benishangul-Gumuz	857	157	118	78	41	37	75.0	13.8	9.1	66.1	52.6	32.8
Dire Dawa	371	177	121	165	29	136	68.4	32.6	44.5	136.4	17.6	59.0
Gambella	381	81	43	32	19	13	52.9	11.3	8.4	74.4	59.4	32.0
Harari	190	155	123	87	8	79	79.6	64.7	45.7	70.7	9.2	56.1
Oromia	25183	3087	2564	1409	776	633	83.1	10.2	5.6	55.0	55.1	29.5
SNNPR	12648	685	443	562	173	389	64.7	3.5	4.4	126.9	30.8	37.3
Somali	4201	221	136	29	8	21	61.7	3.2	0.7	21.3	27.6	14.4
Tigray	2715	598	265	566	444	122	44.3	9.8	20.8	213.6	78.4	64.2
Total (Ethiopia)	60686^a^	7470 ^b^	5696^c^	4530^d^	2424^e^	2106^f^	76.3	9.4	7.5	79.5	53.5	33.9

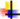
 ^a^: Total estimated number of maternal deaths expected to occur in Ethiopia from 2014 to 2020.

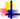
 ^b^: Total identified maternal deaths from other potential data sources, including DHIS-2 and performance reports, in Ethiopia from 2014 to 2020.

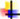
 ^c^: Total notified maternal deaths reported through the PHEM weekly reporting system from 2014 to 2020.

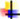
 ^d^: Total reviewed maternal deaths reported through the Maternal Death Reporting Format (MDRF) to the national data hub from 2014 to 2020.

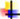
 ^e^: Total community-level maternal deaths reviewed and sent through the Maternal Death Reporting Format (MDRF) to the national data hub after extracting information using Verbal Autopsy (VA) in Ethiopia from 2014 to 2020.

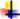
 ^f^: Total facility-level maternal deaths reviewed and reported through the Maternal Death Reporting Format (MDRF) to the national data hub, after extracting information using the Facility-Based Maternal Death Abstraction Format (FBMDAF) in Ethiopia from 2014 to 2020.

**Fig 1:**
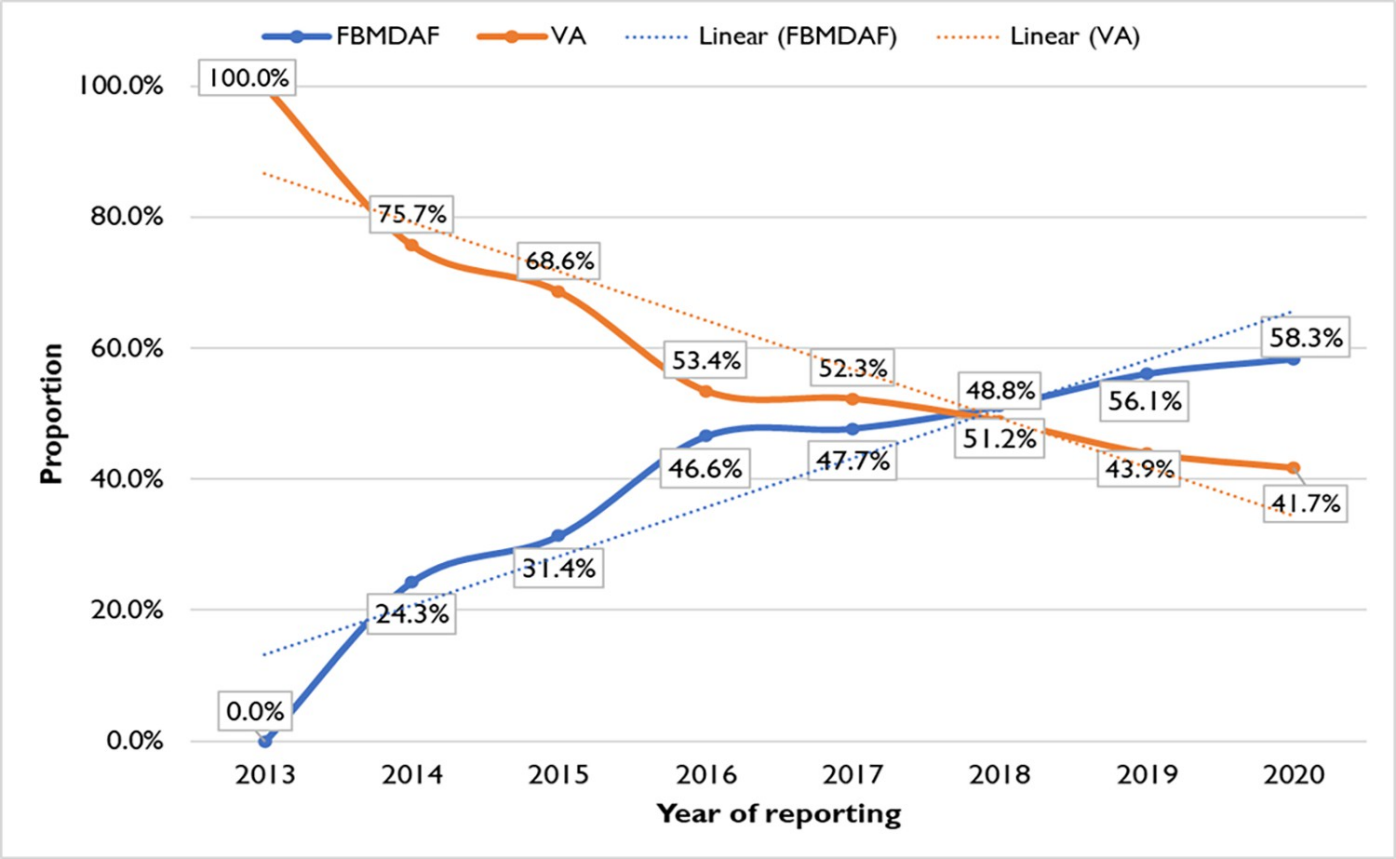
Proportion of reviewed maternal deaths by the source of data extraction from 2013 to 2020 in Ethiopia: FBMDAF (Facility-Based Maternal Death Abstraction Format) and VA (Verbal Autopsy). Case-based reporting using the MDRF (Maternal Death Reporting Format) was initiated in 2013.

The review rate was 79.5%, and regions like Tigray (213.6%), SNNPR (126.9%), and Dire Dawa (136.4%) scored above 100%, indicating a gap in notifying deaths based on available data sources. On the other hand, this shows the functionality of the review committee to review deaths irrespective of their notification status. Moreover, the proportion of reviewed community deaths was 53.5%, with the highest proportion observed in Tigray (78.4%) and Amhara (66.9%), indicating that these regions have better capacity and performance in conducting community surveillance (Table 8). Furthermore, the proportion of community-level reviewed maternal deaths declined from 100% in 2013 to 41.7% in 2020, with a Sen’s slope of -0.5 (95% CI: -0.3 to -0.8, p = 0.003). In contrast, facility-level maternal deaths increased from 0% in 2013 to 58.3% in 2020, also showing a statistically significant increase with a Sen’s slope of 0.5 (95% CI: 0.3 to 0.8, p = 0.003) (Fig 1).

Overall, the MDSRPI was 33.9%, which was declared as low-performing, characterized by low representativeness, completeness, and community engagement. However, only Tigray was identified as a good-performing region, while Amhara, Harari, and Dire Dawa were identified as moderate-performing compared to the remaining regions of Ethiopia (Table 8). Additionally, the detailed notified, reviewed, estimated, and identified maternal deaths from 2014 to 2020 are annexed in **S6-S10 Appendices in S1 File.**

## Discussion

The implementation status of the Maternal Death Surveillance and Response (MDSR) was evaluated using a modified standard tool among 400 health facilities, and the system’s performance was measured using two key performance indicators based on nationally collected data to provide a comprehensive overview of the system. The overall system implementation was deemed suboptimal, with limited capacity to review notified deaths and capture and review community maternal deaths. Additionally, among the surveillance components, the core function had the lowest score compared with the other components of the system. Furthermore, significant variations in the implementation of the system were observed based on region and type of health facility.

Based on the evaluation findings, the score index for the structure of the system was rated as functioning fairly and only partially achieved its expectations of having a well-developed structure. This was because of limitations in the functionality of the review committee, partner mapping, and clear, well-defined strategies. These findings are consistent with those of previous studies [61,62]. The functionality of the review committee was compromised because of a lack of supportive supervision and staff. This has far-reaching repercussions in accurately determining the causes of death and identifying the contributors to maternal deaths [63,64]. Furthermore, engaging partners is considered a parameter of strong leadership for MDSR implementation [65], although it was identified as a weak point in this evaluation. Henceforth, the quality of leadership plays a pivotal role in mobilizing resources from various actors to address critical gaps in supplies and to support community outreach activities related to safe motherhood. In this way, the engagement of external actors generally plays a role in solidifying the system structure [66,67]. In addition, the lack of well-defined strategies or plans to bolster the system has affected its structure, which has implications for the Woreda Health Sector plan. The plan calls for the establishment of a culture of performance management and accountability to include maternal death as one of the main performance indicators [68,69]. Accordingly, the MDSR system is expected to improve the quality of care and support civil registration and vital statistics, which are the top priorities of the country [14,70]. Considering this, the progress made in Ethiopia to expand its usefulness is not yet adequate [71–73]. Overall, the structure of the system varies based on the region, type of health facility, and location (urban vs. rural). These issues should be considered while planning system improvements, along with enhancing political will and commitment, which are of paramount importance in prioritizing the system as a national agenda and fostering the pace and effort needed to meet national targets (i.e., to reduce the maternal mortality ratio (MMR) to 199 per 100,000 live births by 2025). All these efforts are paving the way to achieve global targets set under the Sustainable Development Goals (SDGs) (i.e., reducing the global MMR to less than 70 per 100,000 live births by 2030) [74].

According to the evaluation findings, the core function of the system was rated as ineffective, and the system was unable to function or perform its essential duties. This colossal deficiency in the system has emanated from poor practices in routine data analysis, developing action plans for reviewed deaths, preparing emergency response plans for known major causes of death, assigning causes of death, tracking developed action plans, and providing feedback to lower levels and receiving feedback from higher levels. A similar finding was also revealed in evaluations conducted in Nigeria and Tanzania [75,76]. Routine analysis of MDSR, which includes simple line listing, descriptive statistics (people, place, and time of maternal deaths), and case reviews [77], is not a common practice in Ethiopia, particularly at a lower level of support for routine activities [78,79]. In light of this gap, Ethiopia has made significant investments in establishing a Performance Monitoring Team (PMT) at the facility level as part of its information revolution strategy. These teams aim to encourage routine data analysis and utilization for decision-making, including planning, monitoring, quality improvement, budget allocation, supervision, and feedback [80–82]. However, despite these efforts, the desired outcome has not been achieved in terms of effective utilization of data at the subnational level (i.e., health facilities, districts, and provinces). This is primarily due to infrastructure limitations (e.g., computer and Internet access), negative attitudes toward data, inadequate data quality, an absence of recognition for outstanding performance, insufficient training, and a lack of guidelines and formats [83–86]. Furthermore, at the national level, gaps are observed in conducting in-depth analyses and data triangulation, which are major obstacles to improving the culture of data use [87,88]. To expand on this, in Ethiopia, among the reviewed deaths, 8% had no assigned cause of death [28], indicating the need for computerized analysis of verbal autopsies to be in place [89]. Embedding the Maternal and Perinatal Death Surveillance and Response (MDSR) system within existing health systems is recommended, with strategies that include enhancing Program Management Teams (PMTs), conducting regular review meetings, strengthening routine joint steering committee meetings among regions and partners, improving plan alignment forums, and providing mentoring on data analysis. Additionally, sharing and incentivizing data utilization through regular training, organizing recognition programs, and arranging peer learning sessions are recommended to enhance the utilization of MDSR data [90,91]. To this end, these efforts will have far-reaching implications, from routinely analysing MDSR data to tracking action plans developed based on review findings.

In this evaluation, the score for supporting function was graded as less functional and imbued with extensive shortcomings. These shortcomings include the unavailability of financial resources, computers, implementation manuals, reporting formats (i.e., VA, FBMDAF, MDSR, and identification and notification formats), training and means of communication tools (such as monthly bulletins and annual reports), and regular supportive supervision for catchment areas that were mentioned upfront. This finding concurred with those reported by Cameroon, Kenya, Malawi, and Nigeria, indicating that such challenges fully hinder system implementation [9]. Recognizing the limitations in the capacity of the system, a capacity-building and mentorship program (CBMP) focusing on clinical and public health was established as an advanced version of supportive supervision. This initiative has resulted in improvements in the quality of care and data utilization [92–95]. However, due to the lack of an adequate dedicated budget for these initiatives, full implementation at the national level has not materialized [96]. As a way forward, it is recommended to create a facade of academic-government collaboration, along with building local ownership of the program. These approaches have the potential to be useful in sustaining systems [97]. To address this gap in the availability of reporting formats, there has been an initiative to transform paper-based reporting modalities into electronic reporting. District health information systems (DHIS-2) and community health information systems (CHIS) were introduced to the health system for this purpose [98]. However, these initiatives were not fully optimized due to gaps in leaders’ commitment, resource availability, institutionalization, continued capacity building, communication technology infrastructure, and business continuity plans. Hence, further investment is needed to achieve an acceptable level of implementation and transform the data management process [99–102]. Training, including the Maternal Death Surveillance and Response (MDSR), in preservice and in-service training could ensure its sustainability in the long run [103]. Although training is mandatory for effective implementation, it has not yielded the desired outcomes due to limitations in comprehensive planning, effective post-training supervision, and an implementation training curriculum (lacking elements such as individual learning through problem-solving and case analysis as part of project work and portfolios) [104–106]. To address these gaps, accreditation of training materials and broadening alternative training methods such as e-learning have been implemented [107]. With regard to producing and sharing annual reports, they were not regularly produced and made available publicly to a wider audience, which is also a critical gap at the national level [60]. In summary, strengthening the electronic reporting system, sustaining capacity building and mentorship programs, producing regular annual reports, and implementing a comprehensive training curriculum could be taken as effective measures to enhance the supportive function of the system.

According to the evaluation, the system attribute was rated as functioning fairly well, but it has some limitations that hinder the maintenance of the overall quality of surveillance. These limitations revolve around the attributes of simplicity and acceptability, which are expressed in terms of the limited involvement of health professionals in notifying and reporting deaths, the need for higher-level training and follow-up, and a shortage of computer and reporting tools. Similar findings were reported in evaluations conducted in Zimbabwe and Jordan [108,109]. Concerted efforts are needed to institutionalize Maternal Death Surveillance and Response (MDSR) as a routine practice through engagement with all health professionals using advocacy and considering it a performance indicator. This will facilitate the involvement of all health professionals in the system. As mentioned earlier, digitalizing the system with a business continuity plan and maintaining the Capacity Building and Mentorship Program (CBMP) would play a role in stabilizing and enhancing the system’s simplicity.

The system’s overall readiness to implement MDSR was rated as limited functioning with extensive shortcomings that require further improvement. To objectively assess the system’s status, review rates, and community engagement were used as performance indicators. Nationally, only 79.5% of deaths were reviewed after notification, and among those reviewed, 53.5% were community-level deaths. Accordingly, the Tigray region demonstrated superior performance across all system components and performance indicators, despite the health system being languished in the doldrum by the war in 2020 [110]. Before the conflict, the region had a well-established community health structure that supported community surveillance in identifying and reporting deaths to the next level [111–113]. Moreover, the war also highlighted and evidenced the system’s inadequacies during humanitarian emergencies, necessitating further reconsideration and readjustment to address such crises [114]. On the other hand, the findings highlight that the review process heavily relied on facility deaths, although more than 70% of deaths, including maternal deaths, occur at the community level [115,116]. The findings suggest that having a relatively strong system at the health facility level makes the evidence generated from such a system more applicable to improving the quality of care within health facilities while neglecting community-level factors that are commonly related to health-seeking behavior and access. In this regard, improving the engagement of community health structures led by health extension workers (HEWs) and ancillary structures such as the Women’s Development Army (WDA) could improve community surveillance, which has already proven their method of enhancing health service utilization [117]. However, these programs are facing challenges due to gaps in skills and motivation [118,119]. Addressing these gaps and integrating them well into their routine functions could strengthen the community loop of surveillance and could provide ample evidence to prevent community deaths. Furthermore, there were disparities in death reviews among regions, with the Tigray region outperforming others in terms of reviewed deaths. Nevertheless, gaps were observed, as not all reviewed deaths were reported using the weekly reporting system. In summary, system performance varied significantly by region and type of health facility. Efforts should prioritize improving community surveillance and enhancing each system component to optimize performance.

Generally, according to this evaluation finding combined with the Maternal Death Surveillance and Response Performance Index (MDSRPI), the current MDSR system is not in a position to yield the expected outcomes due to limitations in representativeness, completeness, and community engagement. There is ample evidence indicating that the system is not moving forward to meet its objectives, this indicates that a periodic evaluation of the functionality of the system is required to make necessary adjustments. Furthermore, the findings from the current evaluation clearly indicate the paramount importance of having complementary and supplementary systems that enable fine-tuned recommendations, which can be translated into policy and improved practices. In this regard, the institutionalization of facility-based maternal death audits and reviews, along with a confidential inquiry approach (a detailed review of every single death in comparison to clinical standards and current practices), as well as the enhancement of community-based maternal death reviews with the inclusion of social autopsy, has the potential to influence policy and practice [120–122]. Moreover, the establishment and integration of severe maternal morbidity and emerging pathogen surveillance into routine MDSR will play a seminal role in providing more reliable information, which could shape practice in the continuum of care [123–125]. Additionally, the adaptation of tools and practices of After Action Reviews (AAR) could also play a role in identifying best practices, gaps, and lessons learned, thereby ameliorating subsequent measures [126]. By including the aforementioned reformative measures, the MDSR could be recalibrated to align with the fundamental principles of collaborative surveillance, which call for the triangulation of a panoply of sources of information to facilitate better decision-making [127].

The evaluation has limitations that need to be acknowledged during the interpretation of the findings. (1) An economic evaluation was not included to assess how costly the system is. (2) The evaluation did not consider the perinatal death component of the system, which has peculiar features; thus, not all findings could apply to this component. (3) inability to assess all selected health facilities for various reasons, including inaccessibility due to security.

## Conclusion

Overall, the readiness to implement the MDSR system was suboptimal and not in a position to meet its objectives, compounded by noticeable regional variations. Additionally, not all notified deaths were reviewed, particularly those that occurred at the community level. Furthermore, not all reviewed deaths were notified through the weekly reporting system. Additionally, the system has limitations in terms of representativeness, completeness, and community engagement

Therefore, efforts should focus on engaging political leaders to enhance resource mobilization and prioritize this agenda. Moreover, improving data utilization by strengthening both existing and new monitoring mechanisms, as well as optimizing digitalization efforts, is crucial. Furthermore, implementing algorithm-based cause-of-death assignments, enhancing community surveillance, bolstering the Capacity Building and Mentorship Program (CBMP), contextualizing to humanitarian settings, organizing effective training approaches—including pre-service and in-service training—and conducting periodic evaluations are strongly recommended to reduce variations among regions and types of health facilities and to upgrade the system to the next level.

Alongside the aforementioned recommendations, both local and global actors in MDSR should consider calibrating the system within the framework of collaborative surveillance, which necessitates strengthening the inclusion and integration of severe morbidity and selected pathogens surveillance alongside the confidence-inquiry system. This should be followed by a robust monitoring and learning system by adapting tools and practices from After Action Reviews (AAR) and collaborating with the CRVS system, which could open new doors for the development and transformation of the MDSR system and other event-based surveillance systems.

## Supporting information

S1 File (DOCX)
