# Correction: Implementation status of maternal death surveillance and response system in Ethiopia: Evidence from a national-level system evaluation

**DOI:** 10.1371/journal.pone.0332230

**Published:** 2025-09-10

**Authors:** The PLOS One Staff

There is an error in affiliation 1 for authors Neamin Tesfay, Alemu Zenebe, Zewdnesh Dejene and Henok Tadesse. The correct affiliation 1 is: Centre of Public Health Emergency Management, Ethiopian Public Health Institute, Addis Ababa, Ethiopia.

The publisher apologizes for the error.
