# Supplementary material for: Implementation status of maternal death surveillance and response system in Ethiopia: Evidence from a national-level system evaluation
Source: PLoS One. 2024 Dec 3;19(12):e0312958. doi: 10.1371/journal.pone.0312958 (PMC11614257; doi:10.1371/journal.pone.0312958)
Supplement: S1 File — (DOCX) [file pone.0312958.s001.docx]

**Supplementary documents to**

**Implementation status of Maternal Death Surveillance and Response system in Ethiopia: Evidence from a national-level system evaluation**

Neamin Tesfay ^1*^, Alemu Zenebe ^1^, Zewdnesh Dejene ^1^, Henok Tadesse^1^, Fitsum Woldeyohannes ^2^, Araya Gebreyesus^3^, Amit Arora ^4,5,6,7,^**^8^**

^1^Centre of Public Health Emergency Management, Ethiopian Public Health Institute, Addis Ababa, Ethiopia

^2^ Health Financing Program, Clinton Health Access Initiative, Addis Ababa, Ethiopia

^3^Department of Medical Microbiology and Immunology, College of Health Sciences, Mekelle University, Tigray, Ethiopia

^4^#a School of Health Sciences, Western Sydney University, Penrith NSW 2751, Australia

^5^#b Health Equity Laboratory, Campbelltown, NSW 2560, Australia

^6^ Translational Health Research Institute, Western Sydney University, Campbelltown NSW 2560, Australia

^7^ Discipline of Child and Adolescent Health, The Children’s Hospital at Westmead Clinical School, Faculty of

Medicine and Health, The University of Sydney, Westmead NSW 2145, Australia

^8^ Oral Health Services, Sydney Local Health District and Sydney Dental Hospital, NSW Health, Surry Hills NSW

2010, Australia

**S1 Appendix** - Scope of the National Maternal Death Surveillance and Response Evaluation, National MDSR System Evaluation, Ethiopia


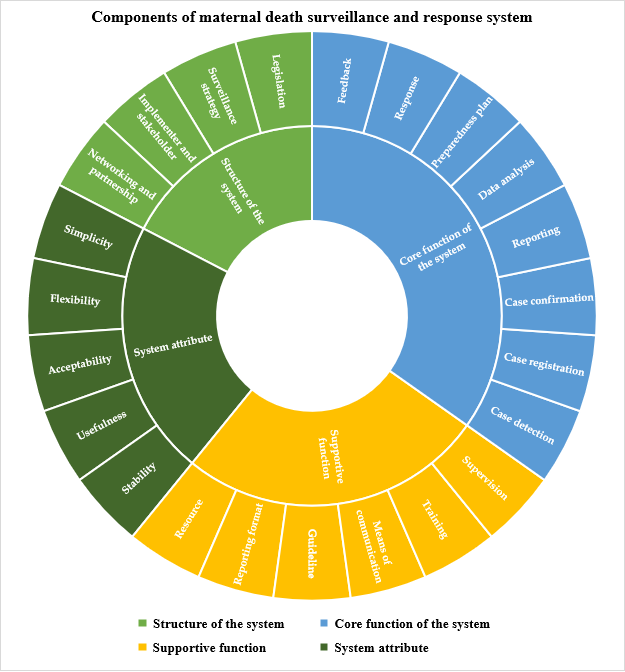


**S2 Appendix:** definition of each indicator for each component of the national maternal death surveillance and response evaluation, national MDSR system evaluation, Ethiopia

| **S. No** | **Component** | **Indicators definition** |
| --- | --- | --- |
| **1** | **Structure of the system** |  |
| **Structure of the surveillance and response system** is defined by legislation, the strategy for implementing activities, the implementers and stakeholders, and how they relate to each other and to the various networks and partnerships | | |
| S1 | Legislation | Assessment of the existence of legislation which supports maternal death notification and reporting |
| S2 | Surveillance strategy | Presence of a strategic and operational plans for implementing and strengthening maternal death surveillance and response |
| S3 | Implementer and stakeholder | Presence of the coordinating platform (maternal death review committee) |
| S4 | Implementer and stakeholder | Check the presence of appropriate composition to review and response deaths |
| S5 | Networking and partnership | The existence of supporting partners or external actors in the coordinating platform |
| S6 | Implementer and stakeholder | Check the functionality of coordinating platform (conducting 4 meetings or more per year) |
| S7 | Implementer and stakeholder | Check availability of assigned surveillance focal |
| 2 | **Core function of the system** |  |
| **Core functions of the system** deals with case detection, case registration, case confirmation, reporting, data analysis and interpretation, and public health response including reports and feedback from the systems to the data providers, stakeholders and decision-makers. | | |
| C1 | Case detection | Ensure the availability of community case definition |
| C2 | Case detection | Ensure the availability of rumor logbook |
| C3 | Case registration | Ensure the availability of registration book |
| C4 | Case confirmation | Ensure the proper utilization of declaimer pledge before reviewing investigated deaths |
| C5 | Reporting | Report reviewed deaths using a weekly reporting format |
| C6 | Data analysis | Conduct routine data analysis |
| C7 | Preparedness plan | Having emergency preparedness plan for the major cause of maternal death |
| C8 | Response | Develop action plan |
| C9 | Response | Track the developed plan (i.e., performance review meeting) |
| C10 | Feedback | Receive feedback for higher-level |
| C11 | Feedback | Provide feedback to the lower level |
| **3** | **Supportive function** |  |
| **Support functions** are those that facilitate implementation of the core functions and included the following trained man power , reporting tools, monitoring and evaluation | | |
| Su1 | Resource | Check the availability allocated financial resource |
| Su2 | Reporting format | Check the availability of maternal death identification and notification format |
| Su3 | Reporting format | Ensure the availability of facility-based abstraction format |
| Su4 | Reporting format | Check the availability of PHEM weekly reporting format |
| Su5 | Reporting format | Check the availability of maternal death reporting format |
| Su6 | Guideline | Check the availability of MPDSR implementation manual |
| Su7 | Means of communication | Produce regular of an annual report |
| Su8 | Means of communication | Ensure the presence of dedicated computer Availability of computer |
| Su9 | Training | Check presence of trained personnel on MDSR |
| Su10 | Supervision | Conduct supportive supervision of MDSR within catchment health facilities |
| **4** | **System attribute** |  |
| **Simplicity** deals with data entry interface, comprehensibility of the variables, comprehensibility of the case definitions, the need for frequent training, and the ease in obtaining the data. | | |
| A1 | Simplicity | Maternal death case definitions are easy to apply |
| A2 | Simplicity | The data sources of MDSR are easy and manageable |
| A3 | Simplicity | Data analysis at the health facility level is easy and manageable |
| A4 | Simplicity | The MDSR system doesn’t take much of my time or not influence my other duties |
| A5 | Simplicity | To work in the system, it doesn’t need high-level training and follow-up |
| A6 | Simplicity | Sending and receiving maternal death reports and distributing the surveillance results are easy |
| A7 | Simplicity | Managing all MDSR-related data and reporting formats is manageable |
| **Flexibility** deals with possibility of adding new variables with minimum cost and effort, the functionality of the system after funding withdrawal, the ability of the restructuring the system to monitor maternal deaths, the ability of the restructuring the system to monitor additional component, the ability of the restructuring the system  to monitor infant deaths, the possibility of integrating. | | |
| A8 | Flexibility | The surveillance system can adapt to the user’s improvement demands like change of case definition, addition to new questions, and changing reporting sources |
| A9 | Flexibility | The system can function easily integrated with other surveillance systems |
| **Acceptability** deals with indicators assessed the user’s willingness to use the MDSR system with and without incentives, their intention to continue using MDSR in the future, and their satisfaction with the system. | | |
| A10 | Acceptability | Responsible bodies to notify/ report suspected maternal deaths from all wards and OPD are sending maternal death reports |
| A11 | Acceptability | All responsible bodies to notify/ report are sending maternal death data in the expected time frame |
| A12 | Acceptability | The information required from the family members or client attendants is acceptable during the investigation process |
| A13 | Acceptability | Health professionals are comfortable to participate in the investigation process |
| A14 | Acceptability | Health professionals are comfortable when they are assigned to death review committees |
| **Usefulness** refers to the relevance of the system to surveillance stakeholders in terms of feeding information for action. | | |
| A15 | Usefulness | The current system can estimate maternal death in the facility/catchment community |
| A16 | Usefulness | The current system can show the trend of maternal death in the facility/ catchment community |
| A17 | Usefulness | The current system can show the modifiable factors to plan for preventive and control strategies against eliminating preventable maternal death |
| A18 | Usefulness | The current system can show the progress and effect of preventive and control methods applied against preventable maternal death |
| A19 | Usefulness | The current system can indicate major causes of maternal deaths in the health facility/ catchment community |
| A20 | Usefulness | The current system can help health facilities improve clinical and ethical practices for quality improvement |
| A21 | Usefulness | The current system can potentially ignite research ideas for further study and investigation |
| A22 | Usefulness | MDSR data or information is always ready for planning purposes |
| **Stability** refers to the reliability (i.e., the ability to collect, manage, and provide data properly without failure) and availability (the ability to be operational when it is needed) of the public health surveillance system. | | |
| A23 | Stability | The system can function with human resource constraints like staff turnover and trained human resource retaining challenges |
| A24 | Stability | The system can easily be operational with busy experts with other jobs |
| A25 | Stability | Shortage of facilities like computers, electricity, and internet service have minimal impact on the system functionality |
| A26 | Stability | Reporting health facilities can work comfortably in the absence of reporting formats |

**S3 Appendix:** score each indicator for each component of the national maternal death surveillance and response evaluation, national MDSR system evaluation, Ethiopia

| **S. No** | **Component** | **Indicators** | **National score(N=400) (Yes) (%)** |
| --- | --- | --- | --- |
| **1** | **Structure of the system** |  |  |
| S1 | Legislation | Mandatory notification of maternal death to the next level | 100.0 |
| S2 | Surveillance strategy | Having a strategy to bolster the implementation of the surveillance | 22.50 |
| S3 | Implementer and stakeholder | Presence of the coordinating platform (maternal death review committee) | 87.25 |
| S4 | Implementer and stakeholder | Appropriate composition of the review committee | 71.50 |
| S5 | Networking and partnership | The existence of supporting partners or external actors in the coordinating platform | 13.00 |
| S6 | Implementer and stakeholder | Functionality of coordinating platform (conducting 4 meetings or more per year) | 20.50 |
| S7 | Implementer and stakeholder | Availability of assigned surveillance focal | 69.75 |
| 2 | **Core function of the system** |  |  |
| C1 | Case detection | Availability of community case definition | 40.50 |
| C2 | Case detection | Availability of rumor logbook | 31.00 |
| C3 | Case registration | Availability of registration book | 70.50 |
| C4 | Case confirmation | Filling out declaimer pledge before reviewing investigated deaths | 47.75 |
| C5 | Reporting | Reporting reviewed deaths using a weekly reporting format | 91.25 |
| C6 | Data analysis | Routine data analysis | 1.50 |
| C7 | Preparedness plan | Emergency preparedness plan for the major cause of maternal death | 24.00 |
| C8 | Response | Develop action plan | 22.25 |
| C9 | Response | Track the developed plan (i.e., performance review meeting) | 16.50 |
| C10 | Feedback | Received feedback for higher-level | 40.00 |
| C11 | Feedback | Providing feedback to the lower level | 29.75 |
| **3** | **Supportive function** |  |  |
| Su1 | Resource | Availability allocated financial resource | 6.50 |
| Su2 | Reporting format | Availability of maternal death identification and notification format | 42.25 |
| Su3 | Reporting format | Availability of facility-based abstraction format | 29.25 |
| Su4 | Reporting format | Availability of PHEM weekly reporting format | 82.50 |
| Su5 | Reporting format | Availability of maternal death reporting format | 48.00 |
| Su6 | Guideline | Availability of MPDSR implementation manual | 16.50 |
| Su7 | Means of communication | Production of an annual report | 32.50 |
| Su8 | Means of communication | Availability of computer | 11.3 |
| Su9 | Training | The presence of trained personnel on MDSR | 38.00 |
| Su10 | Supervision | Conducting supportive supervision of MDSR within catchment health facilities | 36.50 |
| **4** | **System attribute** |  |  |
| A1 | Simplicity | Maternal death case definitions are easy to apply | 90.00 |
| A2 | Simplicity | The data sources of MDSR are easy and manageable | 82.75 |
| A3 | Simplicity | Data analysis at the health facility level is easy and manageable | 82.00 |
| A4 | Simplicity | The MDSR system doesn’t take much of my time or not influence my other duties | 64.25 |
| A5 | Simplicity | To work in the system, it doesn’t need high-level training and follow-up | 44.00 |
| A6 | Simplicity | Sending and receiving maternal death reports and distributing the surveillance results are easy | 76.25 |
| A7 | Simplicity | Managing all MDSR-related data and reporting formats is manageable | 100.00 |
| A8 | Flexibility | The surveillance system can adapt to the user’s improvement demands like change of case definition, addition to new questions, and changing reporting sources | 83.00 |
| A9 | Flexibility | The system can function easily integrated with other surveillance systems | 86.75 |
| A10 | Acceptability | Responsible bodies to notify/ report suspected maternal deaths from all wards and OPD are sending maternal death reports | 72.25 |
| A11 | Acceptability | All responsible bodies to notify/ report are sending maternal death data in the expected time frame | 13.75 |
| A12 | Acceptability | The information required from the family members or client attendants is acceptable during the investigation process | 83.75 |
| A13 | Acceptability | Health professionals are comfortable to participate in the investigation process | 80.25 |
| A14 | Acceptability | Health professionals are comfortable when they are assigned to death review committees | 81.75 |
| A15 | Usefulness | The current system can estimate maternal death in the facility/catchment community | 84.75 |
| A16 | Usefulness | The current system can show the trend of maternal death in the facility/ catchment community | 84.75 |
| A17 | Usefulness | The current system can show the modifiable factors to plan for preventive and control strategies against eliminating preventable maternal death | 88.00 |
| A18 | Usefulness | The current system can show the progress and effect of preventive and control methods applied against preventable maternal death | 90.75 |
| A19 | Usefulness | The current system can indicate major causes of maternal deaths in the health facility/ catchment community | 90.25 |
| A20 | Usefulness | The current system can help health facilities improve clinical and ethical practices for quality improvement | 90.25 |
| A21 | Usefulness | The current system can potentially ignite research ideas for further study and investigation | 86.00 |
| A22 | Usefulness | MDSR data or information is always ready for planning purposes | 87.50 |
| A23 | Stability | The system can function with human resource constraints like staff turnover and trained human resource retaining challenges | 65.75 |
| A24 | Stability | The system can easily be operational with busy experts with other jobs | 58.00 |
| A25 | Stability | Shortage of facilities like computers, electricity, and internet service have minimal impact on the system functionality | 56.25 |
| A26 | Stability | Reporting health facilities can work comfortably in the absence of reporting formats | 42.75 |

**S4 Appendix**: Sample size estimation

The total sample size was computed as $n=n_{health centers}+n_{hospitals}=629$

, where $N_{i}$ is $i^{th}$ population size i= 1, 2 denoting the total number of health centers and Hospitals, $Deff$ is the design effect, considered to be 1.2 for this study. The variance ( $\sigma^{2}$ ) can be obtained from previous studies or pilots. However, the investigators of this study did not do so; instead, approximated it from the variance of a binomial distribution. This represents the combined or pooled variance of the system, indicating that the system’s performance is based on all the indicators. It is estimated as $\hat{\sigma}^{2}=s^{2}$=$\frac{n}{n-1}\hat{p}(1-\hat{p})$, where $\hat{p}=0.5$ is the probability that the system is performing or functioning taking into account the correction for continuity due to approximating the continuous random variable [normal] by a discrete one [ binomial]. Here, $s^{2}$ is sample variance estimating population parameter and for large sample size $\frac{\boldsymbol{n}}{\boldsymbol{n-1}}\boldsymbol{=1}$. Then the total sample size is given by:

$$\boldsymbol{n=}\boldsymbol{n}_{\boldsymbol{1}}\boldsymbol{+}\boldsymbol{n}_{\boldsymbol{2}}$$

**S5 Appendix:** Weight given for component of maternal death surveillance and response in Ethiopia,2020

| **Dimension** | **Weight** |
| --- | --- |
| Structure of the system | 0.25 |
| Core function | 0.25 |
| Supportive function | 0.25 |
| System attributes | 0.25 |

**S6 Appendix**: Estimated maternal deaths in Ethiopia by region from 2014 to 2020

| **Region** | **Year of reporting** | | | | | | | **Total expected death from**  **2014 to 2020** |
| --- | --- | --- | --- | --- | --- | --- | --- | --- |
|  | **2014** | **2015** | **2016** | **2017** | **2018** | **2019** | **2020** |  |
| Addis Ababa | 304 | 313 | 317 | 329 | 327 | 335 | 343 | 2268 |
| Afar | 170 | 180 | 184 | 172 | 184 | 188 | 193 | 1273 |
| Amhara | 1439 | 1524 | 1488 | 1464 | 1519 | 1546 | 1574 | 10552 |
| Benishangul-Gumuz | 113 | 116 | 116 | 128 | 124 | 127 | 131 | 857 |
| Dire Dawa | 49 | 52 | 52 | 52 | 54 | 55 | 57 | 371 |
| Gambella | 50 | 52 | 52 | 56 | 55 | 57 | 59 | 381 |
| Harari | 25 | 24 | 32 | 24 | 27 | 28 | 29 | 190 |
| Oromia | 3364 | 3513 | 3541 | 3577 | 3636 | 3729 | 3824 | 25183 |
| SNNPR | 1698 | 1760 | 1776 | 1809 | 1825 | 1868 | 1912 | 12648 |
| Somali | 560 | 585 | 581 | 605 | 607 | 623 | 639 | 4201 |
| Tigray | 369 | 385 | 385 | 381 | 391 | 398 | 405 | 2715 |
| Total (Ethiopia) | 8159 | 8513 | 8541 | 8601 | 8753 | 8955 | 9163 | 60686 |

- The population data was sourced from central statistics estimates.
- The maternal mortality rate was taken from the UN MMEIG 2017 report, which recorded 401 maternal deaths per 100,000 live births

**S7 Appendix:** Identified maternal deaths from all sources in Ethiopia by region from 2014 to 2020

| **Region** | **Year of reporting** | | | | | | | **Total death identified from all source**  **from 2014 to 2020** |
| --- | --- | --- | --- | --- | --- | --- | --- | --- |
|  | **2014** | **2015** | **2016** | **2017** | **2018** | **2019** | **2020** |  |
| Addis Ababa | 18 | 27 | 22 | 85 | 110 | 87 | 65 | 414 |
| Afar | 11 | 15 | 22 | 57 | 25 | 13 | 45 | 188 |
| Amhara | 33 | 120 | 202 | 373 | 365 | 299 | 315 | 1707 |
| Benishangul-Gumuz | 9 | 2 | 12 | 48 | 24 | 28 | 34 | 157 |
| Dire Dawa | 13 | 12 | 40 | 40 | 21 | 27 | 24 | 177 |
| Gambella | 5 | 7 | 13 | 19 | 12 | 14 | 11 | 81 |
| Harari | 6 | 17 | 10 | 12 | 34 | 36 | 40 | 155 |
| Oromia | 89 | 194 | 335 | 894 | 612 | 513 | 450 | 3087 |
| SNNPR | 24 | 29 | 58 | 247 | 119 | 114 | 94 | 685 |
| Somali | 17 | 14 | 36 | 18 | 23 | 54 | 59 | 221 |
| Tigray | 53 | 75 | 87 | 104 | 92 | 98 | 89 | 598 |
| Total (Ethiopia) | 278 | 512 | 837 | 1897 | 1437 | 1283 | 1226 | 7470 |

**S8 Appendix:** Total reviewed maternal deaths (both community and facility level) in Ethiopia by region from 2014 to 2020

| **Region** | **Year of reporting** | | | | | | | **Total reviewed deaths from 2014 to 2020** |
| --- | --- | --- | --- | --- | --- | --- | --- | --- |
|  | **2014** | **2015** | **2016** | **2017** | **2018** | **2019** | **2020** |  |
| Addis Ababa | 11 | 39 | 25 | 59 | 54 | 48 | 38 | 274 |
| Afar | 1 |  | 10 | 49 | 2 |  | 16 | 78 |
| Amhara | 75 | 144 | 286 | 321 | 207 | 85 | 132 | 1250 |
| Benishangul Gumuz |  | 1 | 10 | 42 | 8 | 8 | 9 | 78 |
| Dire Dawa | 46 | 21 | 44 | 34 | 8 |  | 12 | 165 |
| Gambella |  | 2 | 6 | 16 |  | 7 | 1 | 32 |
| Hareri | 21 | 16 | 3 | 10 | 7 | 12 | 18 | 87 |
| Oromia | 98 | 106 | 192 | 453 | 216 | 216 | 128 | 1409 |
| SNNP | 10 | 72 | 144 | 212 | 86 | 18 | 20 | 562 |
| Somali |  |  |  | 2 |  | 7 | 20 | 29 |
| Tigray | 47 | 90 | 107 | 103 | 94 | 75 | 50 | 566 |
| Total (Ethiopia) | 309 | 491 | 827 | 1301 | 682 | 476 | 444 | 4530 |

**S9 Appendix:** Reviewed community maternal deaths in Ethiopia by region from 2014 to 2020

| **Region** | **Year of reporting** | | | | | | | **Total reviewed community death**  **from 2014 to 2020** |
| --- | --- | --- | --- | --- | --- | --- | --- | --- |
|  | **2014** | **2015** | **2016** | **2017** | **2018** | **2019** | **2020** |  |
| Addis Ababa | 0 | 14 | 2 | 22 | 5 | 4 | 2 | 49 |
| Afar | 1 | 0 | 6 | 25 | 2 | 0 | 7 | 41 |
| Amhara | 73 | 110 | 185 | 203 | 126 | 56 | 83 | 836 |
| Benishangul Gumuz | 0 | 1 | 8 | 21 | 5 | 6 | 0 | 41 |
| Dire Dawa | 7 | 4 | 9 | 9 | 0 | 0 | 0 | 29 |
| Gambella | 0 | 2 | 2 | 12 | 0 | 2 | 1 | 19 |
| Hareri | 3 | 3 | 2 | 0 | 0 | 0 | 0 | 8 |
| Oromia | 97 | 96 | 119 | 239 | 98 | 74 | 52 | 775 |
| SNNP | 10 | 28 | 32 | 67 | 31 | 4 | 1 | 173 |
| Somali | 0 | 0 | 0 | 0 | 0 | 0 | 8 | 8 |
| Tigray | 46 | 79 | 77 | 82 | 66 | 63 | 31 | 444 |
| Total (Ethiopia) | 237 | 337 | 442 | 680 | 333 | 209 | 185 | 2423 |

**S10 Appendix:** Reviewed facility maternal deaths in Ethiopia by region from 2014 to 2020

| **Region** | **Year of reporting** | | | | | | | **Total reviewed facility death**  **from 2014 to 2020** |
| --- | --- | --- | --- | --- | --- | --- | --- | --- |
|  | **2014** | **2015** | **2016** | **2017** | **2018** | **2019** | **2020** |  |
| Addis Ababa | 11 | 25 | 23 | 37 | 49 | 44 | 36 | 225 |
| Afar |  |  | 4 | 24 |  |  | 9 | 37 |
| Amhara | 2 | 34 | 101 | 118 | 81 | 29 | 49 | 414 |
| Benishangul Gumuz |  |  | 2 | 21 | 3 | 2 | 9 | 37 |
| Dire Dawa | 39 | 17 | 35 | 25 | 8 |  | 12 | 136 |
| Gambella |  |  | 4 | 4 |  | 5 |  | 13 |
| Hareri | 18 | 13 | 1 | 10 | 7 | 12 | 18 | 79 |
| Oromia | 1 | 10 | 73 | 214 | 118 | 142 | 76 | 634 |
| SNNP |  | 44 | 112 | 145 | 55 | 14 | 19 | 389 |
| Somali |  |  |  | 2 |  | 7 | 12 | 21 |
| Tigray | 1 | 11 | 30 | 21 | 28 | 12 | 19 | 122 |
| Grand Total | 72 | 154 | 385 | 621 | 349 | 267 | 259 | 2107 |
